# G‑quadruplexes as regulatory platforms for human transcription factors: insights from ChIP-seq data

**DOI:** 10.1186/s12864-026-13217-5

**Published:** 2026-07-29

**Authors:** Karolína Drápalová, Michaela Dobrovolná, Filip Kledus, Jean-Louis Mergny, Václav Brázda

**Affiliations:** 1 Institute of Biophysics, Czech Academy of Sciences, Královopolská 135, Brno, 612 00 Czech Republic; 2 Faculty of Chemistry, Brno University of Technology, Purkyňova 118, Brno, 612 00 Czech Republic; 3 National Centre for Biomolecular Research, Faculty of Science, Masaryk University, Kamenice 5, Brno, 62500 Czech Republic; 4 Laboratoire d’Optique et Biosciences, Ecole Polytechnique, CNRS, Inserm, Institut Polytechnique de Paris, Palaiseau, 91120 France

**Keywords:** G-quadruplex, transcription factors, ChIP-seq, ChIP-Atlas, Gene regulation, Genome-wide analysis

## Abstract

**Background:**

G-quadruplexes (G4s) are non-canonical DNA structures with important regulatory functions. While several transcription factors have been shown to interact with G4s, a comprehensive understanding of this interaction network remains elusive.

**Results:**

Here, we integrated genome-wide predictions of highly stable G-quadruplex sequences with 32,817 ChIP-seq datasets from ChIP-Atlas to systematically map transcription factors and transcription-associated chromatin proteins linked to G4-rich regions in the human genome. Highly stable G4 motifs are non-randomly distributed, showing strong enrichment in gene-dense chromosomes, at promoters, regulatory regions, and repeat elements. Integration with transcription factor binding profiles revealed a broad spectrum of G4-associated proteins, including established interactors such as STAT3, TP53 and CTCF, and the transcription-associated chromatin regulator BRD4, as well as previously unrecognized candidates such as REST, NR3C1, FLI1, and HSF1. Unexpectedly, only a minority of transcription factors were consistently depleted from G4 regions.

**Conclusions:**

Our results indicate that G-quadruplex-prone sequences represent a common genomic feature associated with a number of human transcription factors and chromatin-associated regulatory proteins and support a model in which G4-rich regions act as selective regulatory scaffolds shaping transcription factor occupancy and gene regulation.

**Graphical abstract:**

**Figure:**
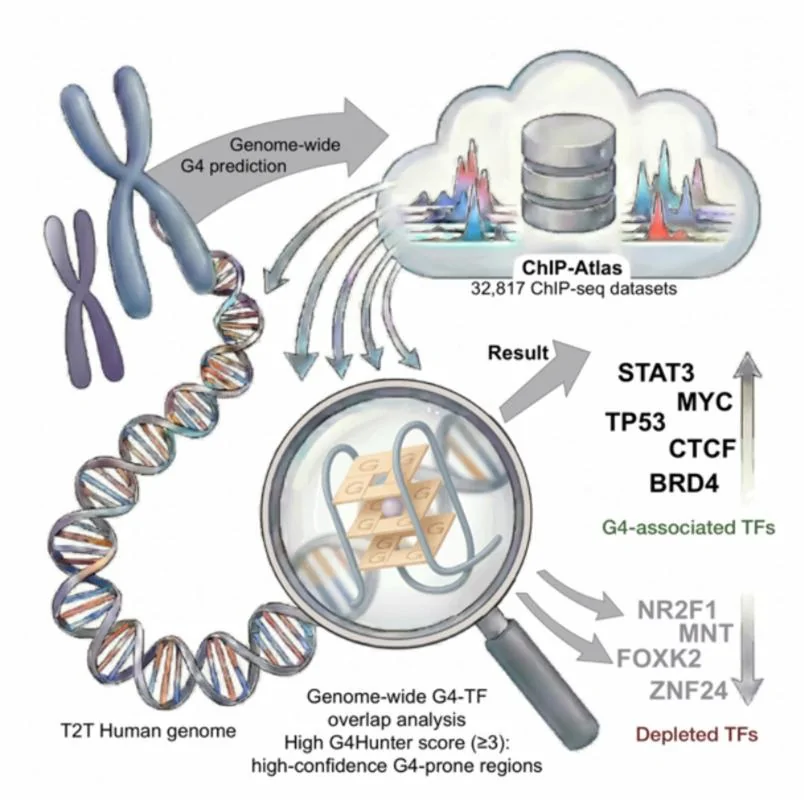

**Supplementary Information:**

The online version contains supplementary material available at https://doi.org/10.1186/s12864-026-13217-5.

## Background

Guanine-rich DNA sequences naturally fold into four-stranded secondary structures called G-quadruplexes (G4s), which consist of stacked guanine tetrads stabilized by Hoogsteen hydrogen bonds and monovalent cations [1]. These structures can form intramolecularly or intermolecularly, adopting parallel, antiparallel or hybrid topologies, depending on sequence and physiological conditions [2]. G4s have attracted considerable attention because of their regulatory roles in biologically important genomic regions across various organisms [3, 4], including humans [5–7]. G4s are involved in several critical cellular processes, such as gene transcription, translation, DNA replication, and genomic stability. While G4 structures can form in both DNA and RNA, their stability and biological roles vary depending on the chemical nature of the molecule – DNA G4s and RNA G4s differ in sugar backbone composition, folding constraints, and cellular localization [8]. Genome-wide analyses have shown that potential G4 motifs are non-randomly distributed across the genome and are significantly enriched in promoters [9, 10]. G4 motifs are also frequently found near CpG islands – genomic regions characterized by high GC content and elevated CpG dinucleotide density relative to the rest of the genome – which commonly overlap transcription start sites at the 5′ ends of genes [11]. In addition, G4s occur in 5′-untranslated regions (5′UTRs) of mRNAs, where RNA G4 structures can modulate translation efficiency, as demonstrated for the NRAS gene [12]. G4 motifs were also found enriched at replication origins [13], and in telomeric DNA, which naturally contains G-rich repeats predisposed to G4 formation [1, 14].

Transcription factors (TFs) are major regulators of gene expression, controlling transcription initiation, elongation, and chromatin organization. Their genomic occupancy has traditionally been understood through the recognition of short linear double-stranded DNA motifs; however, accumulating evidence shows that TF binding is also shaped by higher-order DNA structures and the surrounding chromatin context [15, 16]. Numerous studies have demonstrated that several non-B DNA conformations (including Z-DNA, cruciforms, triplexes, and G4s) can influence or regulate TF activity [17, 18]. Several studies also show that G4s are important promoter elements [9, 19], allowing nucleosome exclusion and facilitating pause–release by RNA polymerase II [19]. Overall, G4s contribute to transcription regulation and modulation of promoter usage [20, 21], including alternative promoter choice [20]. G4 motifs abundance were shown to be highly enriched in several oncogenes such as MYC [22], KRAS [23], B-MYB [24] or VEGF [25]. Experimental studies demonstrate that G4 formation can impede RNA polymerase II progression or restrict access to duplex-binding TFs [22, 23, 25], while in other contexts, G4s create structure-specific docking sites for regulatory proteins [26]. These observations illustrate that G4 folding adds an additional layer of regulation on top of the classical promoter architecture.

Understanding TF–G4 interactions is particularly important because the outcome is highly context dependent: promoter G4s can either facilitate or inhibit TF binding [22, 23], thereby modulating gene expression. For instance it was shown that G4 stabilization by small-molecule G4-ligands of the MYC promoter enhances the binding of repressive G4-interacting proteins, thereby preventing the assembly of activating TFs and leading to transcriptional repression [22]. In contrast, the G4 structure within the KRAS promoter can hinder the binding of TFs required for activation and TF–G4 interactions can either repress or activate gene expression, depending on whether the promoter G4 is folded or unwound [23]. Recently, a growing number of proteins have been identified that specifically interact with G4 structures as shown in G4IPDB database which compiles over 200 experimentally validated G4–protein interactions across species, providing a comprehensive resource for studying the G4 interaction network [27]. A well-characterized example is Nucleolin, which recognizes and stabilizes most G4s, including the MYC promoter G4, displacing activating factors and repressing transcription [28, 29]. Beyond transcriptional regulation, G4–protein interactions may play roles in post-transcriptional regulation and intercellular communication. For instance, recent work showed that G4-binding proteins modulate microRNA loading into small extracellular vesicles, linking G4 biology to aging and vesicle-mediated signaling [30]. Moreover, both FUS [31] and ALYREF [32] have been shown to bind G‑quadruplex structures, and this interaction contributes to the regulation of microRNA distribution. These and other G4-binding proteins contain the so called RGG or NIQI motif [33, 34]. However G4IPDB database contains also proteins without this motif pointing to the various mechanisms of protein interaction with G4s [27].

Large-scale chromatin immunoprecipitation followed by sequencing (ChIP-seq) datasets now provide a powerful resource for genome-wide analyses of TF binding. However, the activity of many TFs cannot be fully understood without considering the influence of G4 structures on their binding behavior. Defining TF binding at G4 motifs is therefore critical for understanding how non‑canonical DNA conformations shape promoter regulation and genome function. Genome‑wide analysis of G4-dependent regulation requires methods that capture protein–DNA interactions in vivo, with ChIP‑seq remaining the most widely used approach for mapping TF occupancy, histone marks, and other regulatory factors. The latest generation of ChIP-Atlas was specifically developed to take advantage of this wealth of information by applying a unified re-processing pipeline to all publicly available datasets [35]. Despite growing evidence that G4s shape TF binding and gene regulation, it remains unclear which TFs co-localize with promoter G4s on a genome-wide scale. This raises a central question: *can large-scale analysis of ChIP-seq data be used to identify previously unrecognized TFs associated with G4-prone motifs?* To address this, we systematically analyzed uniformly processed ChIP-seq datasets from ChIP-Atlas 3.0 [35] to determine which proteins – including TFs and chromatin-associated regulators – show genome-wide co-localization with G4-prone motifs.

## Results

To systematically investigate TF associations with G4-forming sequences on a genome-wide scale, we analyzed large-scale ChIP-seq datasets that capture protein–DNA interactions in cells. Specifically, we used uniformly processed ChIP-seq experiments from ChIP-Atlas 3.0, which provides a comprehensive and comparable collection of datasets across human and model organisms [35].

### Prediction of G4 structures in the human genome by G4Hunter algorithm

**Fig. 1:**
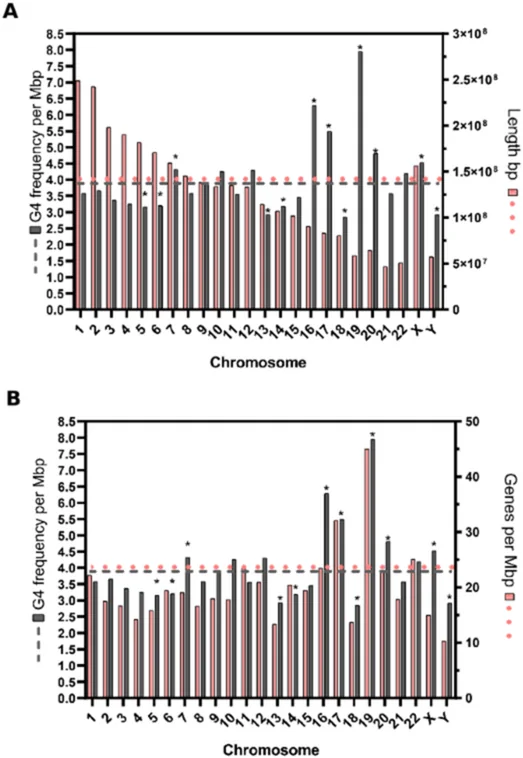
**A** Chromosome lengths and stable G4 densities across the human genome. Each column represents a chromosome and shows the G4 frequency and length calculated for that chromosome using a G4Hunter threshold of 3.0, to pick the most likely and stable quadruplexes. Dashed and dotted lines indicate the median values for G4 density and chromosome length, respectively. Asterisk (*) notations indicate values outside the interquartile range (IQR) for G4 frequency, Q1 = 3.21, Q3 = 4.31 (per Mbp). **B **Correlation between G4 frequency and gene density across human chromosomes. G4 frequency per Mbp (gray bars, left axis) and gene density per Mbp (pink bars, right axis) are shown for each chromosome. Dashed lines indicate genome-wide averages. Asterisks indicate chromosomes with G4 frequency outside the IQR (descriptive outliers). G4 density broadly parallels gene density, with gene-rich chromosomes such as 19 and 17 showing the highest G4 frequencies. However, chromosome 16 exhibits disproportionately high G4 density relative to its gene content, while chromosome Y shows near-average G4 frequency despite exceptionally low gene density, suggesting that additional sequence-compositional factors beyond gene density contribute to regional stable G4 formation

Predictions were made with the G4Hunter algorithm [10], using a highly stringent threshold of ≥ 3.0, thereby selecting high-confidence G4-prone sequences with the strongest G4-forming potential. G4Hunter is one of the most extensively validated G4 prediction algorithms, with quantitative links between score thresholds and experimentally confirmed G4 stability [10, 36]. Importantly, the G4Hunter scoring algorithm explicitly incorporates local guanine richness and sequence skewness, thereby inherently accounting for local GC composition and GC skew in its predictions. Analysis of positive hits revealed an overall frequency of 4.01 G4 motifs/Mbp for the whole human genome. Median value of G4 motifs frequency was 3.58 G4 motifs/Mbp. When considering individual chromosomes, G4 motifs densities varied, reflecting differences in genomic composition and feature distribution (Fig. 1A). For example, chromosomes 9 and 22 exhibited patterns similar to the genome-wide trend, with G4 motifs frequencies of 3.91 and 4.19 respectively. In contrast, chromosome 19 exhibited the highest G4 motifs density (7.95 G4 motifs/Mbp), followed by chromosome 16 (6.29 G4 motifs/Mbp) and chromosome 17 (5.49 G4 motifs/Mbp). This is consistent with the well-documented exceptionally high gene density and GC-rich nature of chromosome 19 [37]. Conversely, chromosomes 18 and Y displayed significantly lower densities (2.85 and 2.92 G4 motifs/Mbp, respectively), with chromosome 13 showing a similarly low value (2.93 G4 motifs/Mbp).

Comparison with gene density per chromosome (Fig. 1B) reveals that G4 frequency broadly parallels gene content; however, chromosome 16 exhibits disproportionately high G4 density relative to its gene density, while chromosome Y shows near-average G4 frequency despite exceptionally low gene content, suggesting that additional sequence-compositional factors contribute to regional G4 formation. Results highlighting gene density and G4‑motif frequencies that fall outside the interquartile range (IQR) are provided in Supplementary material 01.

### Genome-wide positioning of G4 by annotation

**Fig. 2:**
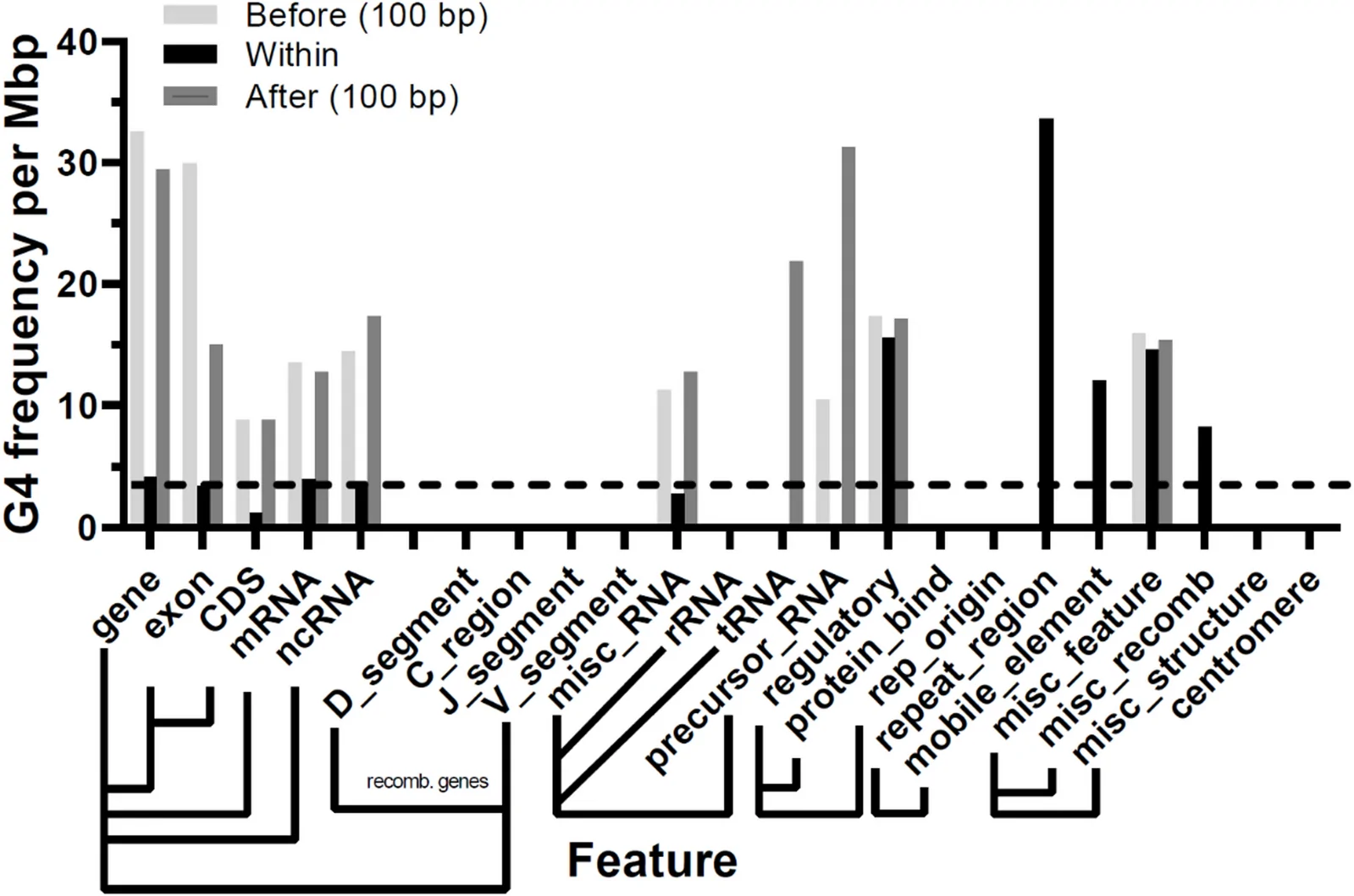
Stable G4 frequencies according to location within (black) annotated human genomic features, as well as 100 bp upstream (light grey) and 100 bp downstream (dark grey) of these regions. The dashed line represents the average G4 frequency across all chromosomes. G4 density was normalized per Mbp. Features on the X-axis are sorted to reflect their hierarchical and partially nested relationships: gene-level annotations (gene, exon, CDS, mRNA, ncRNA) are shown first, followed by immunoglobulin gene segments (D_segment, C_region, V_segment), RNA-related features (misc_RNA, tRNA, precursor_RNA, rRNA), and other functional elements. “precursor_RNA” refers to primary RNA transcripts prior to processing (pre-mRNA). Note that annotations are not fully independent – for example, exons and CDS are subsets of gene annotations. This figure provides a structural genomic context for G4 localization based on genomic annotations and does not represent a functional or expression-resolved classification of genes

To investigate the genomic distribution of G4 motifs in the human genome, we analyzed their frequency across various genomic features annotated in the NCBI reference dataset. For each feature, G4 density was calculated in three separate regions: 100 base pairs upstream (“*before*”), within the element itself (“*within*”), and 100 bp downstream (“*after*”) – Fig. 2, G4 motifs are not evenly distributed across the genome. The most pronounced abundance of these appeared *within* repeat regions, where G4 density approached 35 motifs per Mbp, roughly 9× times higher than the genome-wide average (~ 4 G4s/Mbp, shown by the dashed line). Increased G4 levels were also significant around precursor RNA, especially downstream of the feature, where frequency exceeded 30 G4s per Mbp. G4-prone sequences are also strongly abundant *before* and *after* genes, exons, mRNA regions pointing to their regulatory role in promoters, introns and in UTR regions. Moreover, the strong G4-prone sequences abundance was also both *within* and in proximity of regulatory regions, downstream of tRNA genes (around 5× above average). By contrast, some regions such as centromeres and rRNA sequences were strongly depleted for G4-prone sequences.

### TF–G4 association profiling using ChIP-seq data

We next explored the association of G4-forming regions with large-scale ChIP-seq datasets. We used a collection of 32,817 ChIP-seq experiments from the ChIP-Atlas, involving a wide variety of TFs. As the dataset includes canonical TFs as well as chromatin-associated regulatory proteins, not all entries represent sequence-specific TFs. In total, our combined analysis included over 352 million ChIP-seq peaks for 1846 unique proteins, of which around 14 million intersected with predicted G4 regions. To narrow the focus to high-confidence associations, we applied a stringent statistical significance threshold (*p* < 0.001), which yielded a refined set of 26,288 statistically significant results. These results collectively involved 1,669 unique proteins, encompassing both proteins enriched at and depleted from G4-forming sequence–associated regions.

**Fig. 3:**
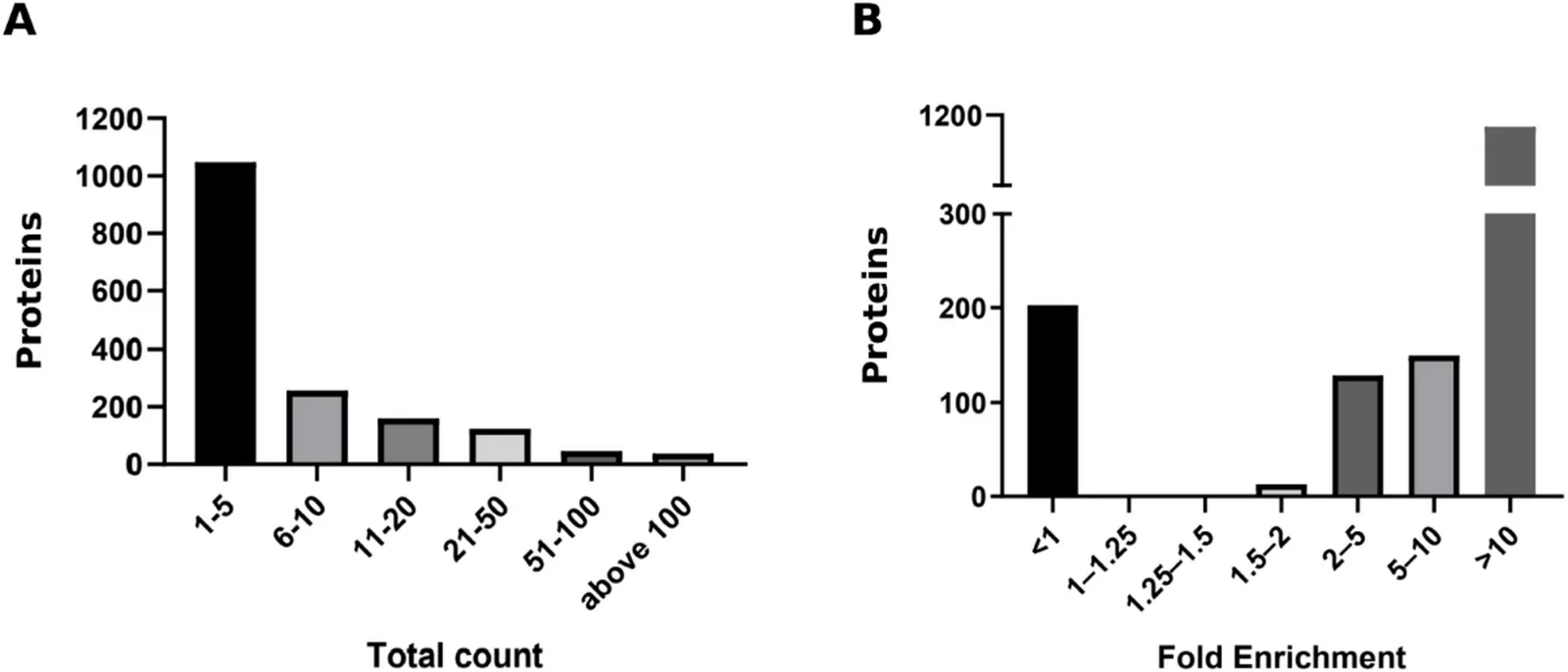
Overview of ChIP-seq dataset coverage and enrichment distribution across all transcription-associated proteins analyzed (*n* = 1,669), including canonical TFs and chromatin-associated regulators. **A** Distribution of proteins according to the number of ChIP-seq experiments in which they were detected. Proteins were grouped into intervals based on total ChIP-seq experiment counts (*n* = 1,669), revealing a progressive decrease in protein frequency with increasing dataset coverage. **B** Distribution of proteins according to fold enrichment (FE) values derived from ChIP-seq experiments. Proteins were classified into defined FE intervals reflecting depletion (< 1), weak (1.0–1.25), moderate (1.25–2.0), strong (2.0–5.0), very strong (5–10) and extremely strong enrichment (> 10)

While the total number of ChIP-seq experiments reflects dataset coverage (Fig. 3A), fold enrichment provides a measure of the relative strength and direction of association with G4-prone sequences (Fig. 3B). As shown in Fig. 3A, the majority of proteins were detected in a limited number of ChIP-seq experiments (1–5 datasets), indicating that most proteins are supported by a small number of datasets. In contrast, the distribution of fold enrichment values (FE) (Fig. 3B) shows a clear separation of proteins into depleted and strongly enriched groups, consistent with the applied significance filter. While a fraction of proteins shows depletion (FE < 1), the dominant feature of the distribution is the high number of proteins with strong (FE 2–5), very strong (FE 5–10) and extremely strong enrichment (FE > 10).

The set of 1,669 proteins was further separated into groups enriched or depleted in G4‑forming sequences based on their FE values. Overall, 1,464 proteins exhibited enrichment (FE > 1), whereas only 205 showed depletion (FE < 1). This means that 88.7% of all TF proteins listed in the ChIP‑Atlas are associated with G4‑forming sequences. When applying a stricter 2x enrichment, 1,437 proteins remain enriched, compared with 201 proteins exhibiting 2x depletion. Very strong enrichment (FE ≥ 5) was observed for 1,311 proteins. An even more selective subgroup displayed extreme enrichment (FE ≥ 10). In total, 1,165 proteins reached this threshold, indicating a highly preferential association with G4-prone sequences. On the opposite end, we found extreme (> 10x) depletion of 151 proteins. These analyses identify proteins with the strongest positive or negative association with G4-prone sequences. Furthermore, a substantial proportion of proteins showed highly consistent enrichment across ChIP‑seq datasets. Out of the complete set of 1,669 identified proteins, 1,020 proteins were enriched in G4-forming sequences in 100% of experiments in which they appeared. When considering proteins enriched in at least 90% of experiments, this number increased to 1,146 proteins, and further to 1,269 proteins at the ≥ 75% enrichment threshold. These observations indicate that the majority of G4‑associated proteins display robust and reproducible enrichment across independent datasets, indicating a high level of consistency across independent datasets.

For each protein, enrichment was evaluated using FE values provided by the ChIP-Atlas resource. The total count corresponds to the number of ChIP-seq datasets available for a selected protein. The enriched and depleted counts indicate the number of datasets in which the protein showed enrichment at G4-prone sequences, respectively. The enrichment percentage (Table 1**)** represents the proportion of datasets with FE > 1 relative to the total count. The “Enriched > 10 count” denotes the number of datasets in which enrichment exceeded a tenfold threshold (FE ≥ 10), reflecting exceptionally strong and consistent association with G4-forming regions. To spotlight proteins with the most consistent associations, we focused on those detected in at least 80 distinct ChIP-seq datasets and showing enrichment in 90–100% of cases (Table 1). Applying these criteria helped reduce background variability and sharpened the detection of proteins with strong, reproducible co-localization patterns. The resulting subset included a mix of TFs, chromatin regulators, and well-characterized DNA-binding proteins that frequently colocalize with G4-forming genomic regions. Interestingly, some proteins demonstrated both enrichment and depletion across different loci, indicating that their association patterns may be modulated by local genomic context rather than G4 structures alone. Among the most prominently enriched factors was STAT3, which was enriched in 100% of its datasets, ranking as the top-performing protein in the dataset. In addition, TP53, BRD4, MYC and CTCF were identified as four other highly enriched proteins, each showing enrichment levels above 95% and ranking among the top four proteins with the highest number of datasets in which the enrichment signal clearly exceeded the background threshold (Enriched > 10 count). These TFs are well-known for their roles in transcriptional regulation and genome architecture, making them particularly relevant candidates for exploring functional associations with G4-forming regions. Their frequent presence near G4s reinforces the biological relevance of these non-canonical DNA structures in key regulatory pathways. A complete list of proteins, grouped by enrichment status, can be found in Supplementary Material 02.

**Table 1:** ChIP-seq enrichment: Proteins present in more than 90% of identified G4-forming sequences (proteins with ≥ 10 ChIP-Atlas entries) with Total count above 80. Total count indicates the number of ChIP-seq datasets available for a given protein. Enriched and depleted counts correspond to the number of datasets showing Enrichment (FE > 1) or Depletion (FE < 1) at G4-prone sequences, respectively. Enrichment (%) represents the fraction of datasets with FE > 1 relative to the total count. Enriched > 10 (%) indicates the proportion of enriched datasets (FE > 1) in which fold enrichment exceeded tenfold (FE ≥ 10). The table encompasses both canonical TFs and transcription-associated chromatin proteins; proteins were not stratified by TF classification. The “G-quadruplex” entry corresponds to ChIP-seq experiments performed with the BG4 antibody, which directly recognizes G-quadruplex DNA structures

Protein	Total count	Enriched count	Depleted count	Enriched > 10 count	Enrich-ment	Enriched > 10 (%)
G-quadruplex	147	147	0	145	100.0%	98.6%
STAT3	127	127	0	76	100.0%	59.8%
LMNB1	85	84	1	82	98.8%	97.6%
STAT1	85	84	1	53	98.8%	63.1%
FLI1	83	82	1	25	98.8%	30.5%
REST	120	118	2	100	98.3%	84.7%
MYCN	118	116	2	72	98.3%	62.1%
RUNX1	154	151	3	82	98.1%	54.3%
YY1	86	84	2	37	97.7%	44.0%
BRD2	113	110	3	49	97.3%	44.5%
HIF1A	112	109	3	87	97.3%	79.8%
TP63	94	91	3	46	96.8%	50.5%
MYC	300	290	10	184	96.7%	63.4%
SUPT5H	87	84	3	39	96.6%	46.4%
JUN	170	164	6	88	96.5%	53.7%
SUZ12	165	159	6	115	96.4%	72.3%
CTCF	1932	1861	71	218	96.3%	11.7%
HSF1	105	101	4	95	96.2%	94.1%
TP53	262	252	10	232	96.2%	92.1%
SMC1A	97	93	4	20	95.9%	21.5%
BRD4	720	690	30	431	95.8%	62.5%
KDM1A	116	111	5	88	95.7%	79.3%
RNF2	169	161	8	111	95.3%	68.9%
RAD21	431	410	21	82	95.1%	20.0%
STAG1	97	92	5	13	94.8%	14.1%
ESR1	1052	997	55	511	94.8%	51.3%
PGR	132	125	7	86	94.7%	68.8%
KMT2A	111	105	6	39	94.6%	37.1%
SPI1	180	170	10	42	94.4%	24.7%
MED1	228	214	14	135	93.9%	63.1%
HOXB13	94	88	6	21	93.6%	23.9%
EZH2	197	184	13	138	93.4%	75.0%
EP300	347	324	23	187	93.4%	57.7%
NR3C1	401	374	27	222	93.3%	59.4%
RELA	268	249	19	153	92.9%	61.4%
CEBPB	131	121	10	29	92.4%	24.0%
GATA2	84	76	8	35	90.5%	46.1%

**Fig. 4:**
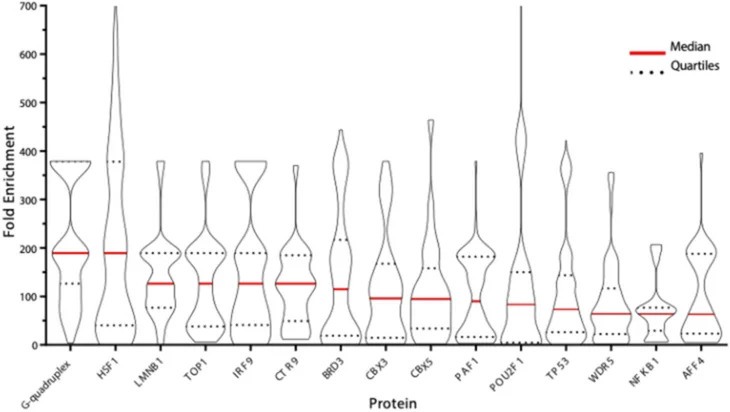
Top 15 ChIP‑seq proteins most strongly enriched in G4‑associated regions. The figure shows the 15 proteins with the highest median fold enrichment within G4‑associated regions, selected from factors represented by ≥ 25 ChIP‑Atlas datasets and detected at least in 90% of G4 samples. Proteins are ordered from left to right by decreasing median fold enrichment. Violin plots display the distribution of fold‑enrichment values; dashed lines denote the IQR, and the central red line indicates the median. The dataset includes both canonical TFs and transcription-associated chromatin proteins; TF-specific stratification was not applied

Out of all proteins profiled in at least 80 independent ChIP-seq datasets, 37 met the predefined criteria of showing enrichment in 90–100% of cases (Table 1). Unsurprisingly, the highest enrichment was observed for the “G-quadruplex” entry itself (147 datasets, enriched in 100%), which corresponds to ChIP-seq experiments performed with BG4 anti-G4 antibody, that directly recognize G-quadruplex DNA structures [38]. This result confirms that BG4 ChIP‑seq datasets consistently show enrichment at predicted G4 regions, serving as an internal positive control for the enrichment analysis. STAT3 was enriched in 100% of its 127 datasets, with 76 of these datasets showing extremely strong enrichment (FE ≥ 10). Several additional proteins were also enriched in a high proportion of their datasets. STAT3 was enriched in 100% of its 127 datasets, with 76 of these datasets showing extremely strong enrichment (FE ≥ 10). Several additional proteins were also enriched in a high proportion of their datasets. TP53, for instance, was enriched in 96.2% of its 262 datasets, and 92.1% of these showed extreme enrichment (FE ≥ 10), indicating a robust and consistent association across diverse conditions. CTCF was enriched in 96.3% of its 1,932 datasets, although only a small fraction reached extreme enrichment (11.7%). MYC and BRD4 combined frequent enrichment (96.7% and 95.8%) with a high proportion of extremely enriched datasets (63.4% and 62.5%). Extreme enrichment was also common for EZH2 (75.0%), NR3C1 (59.4%), EP300 (57.7%), and ESR1 (51.3%). Other proteins were enriched also in majority of ChIP-seq experiments, however only a smaller part of experiments shows this extreme 10-times or more enrichment, such as FLI1 (detected in 83 datasets, 25x above 10-times enrichment) and STAG1 (present in 97 datasets, 13 above 10-times enrichment). Figure 4 depicts the 15 proteins with the highest median fold enrichment across G4‑associated loci, selected on the basis at least 25 independent ChIP‑Atlas entries and abundance in at least 90% of experiments.

In addition to profiling enriched proteins, we also examined those that were consistently depleted at predicted G4-forming sites. Table 2 shows proteins with at least 10 ChIP-seq datasets and evidence of depletion in over half of the experiments, based on fold-enrichment metrics. The group includes several TFs and chromatin-related proteins, such as GATA4, HMGA1, and MNT. Across the analyzed datasets, these factors showed reduced presence at G4-associated loci.

**Table 2:** Depletions of ChIP-seq proteins: in more than 50% of identified G4-forming sequences (proteins with ≥ 10 entries in the ChIP-Atlas). Total count indicates the number of ChIP-seq datasets available for a selected protein. Enriched and depleted counts correspond to the number of datasets showing enrichment (FE > 1) or depletion (FE < 1) at G4-prone sequences, respectively. Depletion (%) represents the fraction of depleted datasets relative to the total count

Protein	Total Count	Enriched Count	Depleted Count	Depletion
GATA4	21	10	11	52.4%
Crotonyllysine	11	5	6	54.5%
NKX2_1	16	7	9	56.3%
CTBP1	14	6	8	57.1%
HMGA1	19	7	12	63.2%
NR2F1	11	4	7	63.6%
MNT	12	4	8	66.7%
FOXK2	11	2	9	81.8%
ZNF24	18	0	18	100.0%

**Fig. 5:**
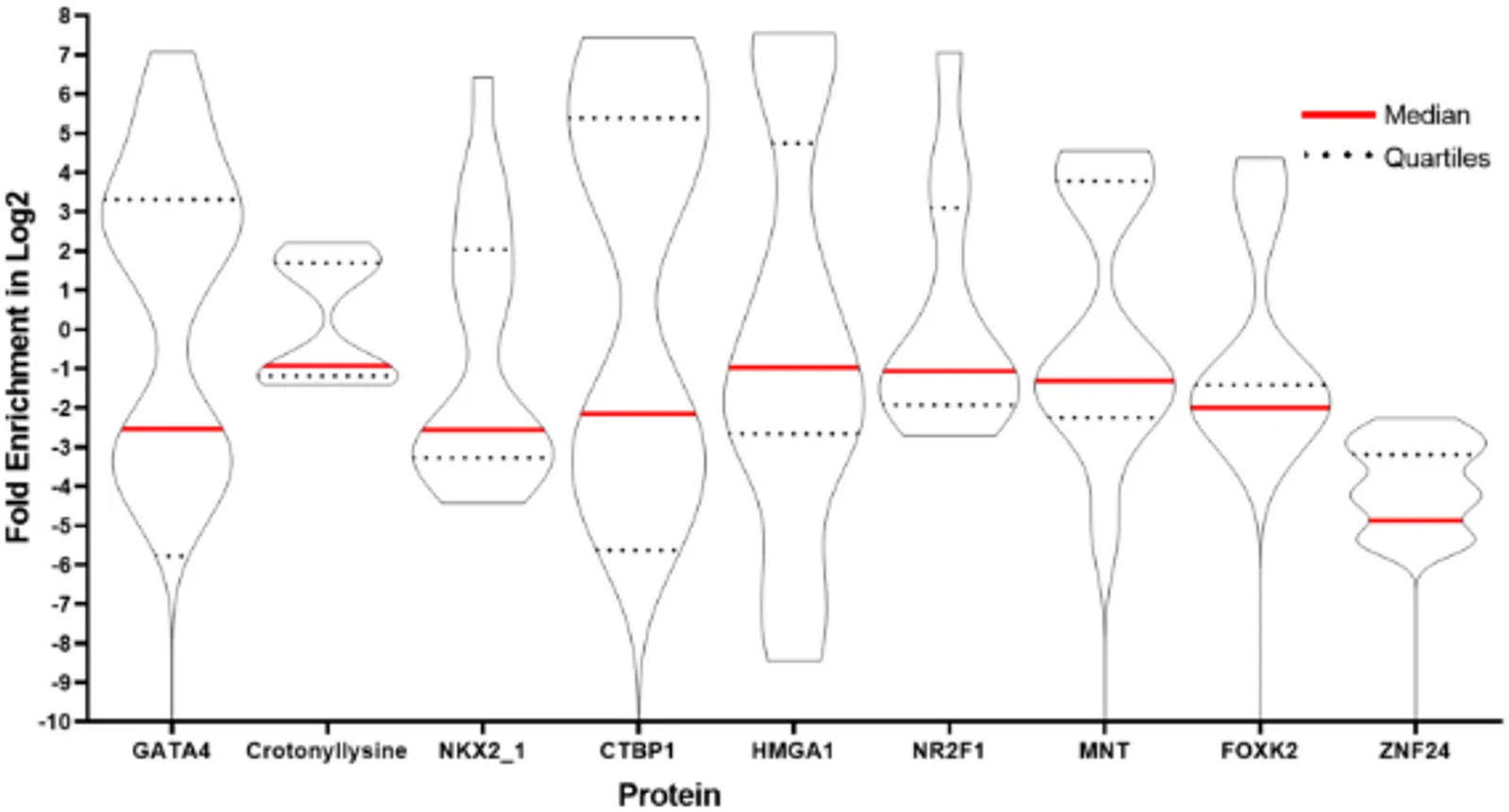
ChIP-seq proteins most strongly depleted in G4-associated regions. Shown are proteins with more than 10 ChIP-Atlas entries that showed depletion (FE < 1) in more than 50% of ChIP-seq datasets for a given protein, ordered from left to right by decreasing fold enrichment. The violin plot displays the distribution of fold-enrichment values on a logarithmic scale. Dashed line indicates the IQR, the red central line denotes the median

Only 9 proteins met the criteria for depletion, defined as appearing in G4-associated regions in no more than half of their ChIP-seq datasets (minimum of 10 experiments) (Fig. 5). Here, depletion is defined as FE < 1 in more than 50% of ChIP-seq datasets for a given protein – reflecting below-chance overlap with G4-forming sequences – not reduced co-localization frequency with individual G4 loci. Among them, ZNF24 was the only protein depleted in all 18 of its datasets, showing no enrichment (FE > 1) in any of them. Except for ZNF24, all depleted proteins showed enrichment (FE > 1) in at least one ChIP-seq dataset, but were classified as depleted because the majority of their datasets showed FE < 1, indicating that their overall association with G4 regions is below what would be expected by chance. After ZNF24, the depletion rates declined sharply. FOXK2 was depleted in 81.2% of cases, followed by MNT (66.7%) and HMGA1 (63.2%). GATA4 was near the cutoff point, each showing nearly balanced numbers of enriched and depleted entries.

To provide a clearer view of signal depletion among G4-associated proteins, Fig. 5 shows the distribution of FE values for proteins identified as depleted in G4-forming regions. The protein ZNF24 showed a median enrichment value of zero, confirming its complete absence from G4-associated regions. Similarly, FOXK2 and Crotonyllysine had medians close to zero, suggesting minimal co-localization with G4 motifs. Only occasionally higher FE values were observed in individual experiments of other depleted proteins, these cases were infrequent and did not affect the overall classification of these proteins as depleted, which was based on the majority of available samples in ChIP-seq datasets. Compared to the broader set of enriched factors, the number of consistently depleted proteins is noticeably smaller.

As a control, we performed the same enrichment analysis using input‑control datasets from the ChIP‑Atlas. Input controls appeared enriched in only 54.5% of cases across all proteins: among 17,887 input datasets, 9,744 appeared enriched and 8,143 depleted. However, in contrast to the 80.1% of significant enrichments observed in the TF dataset overlay with G4–forming sequences, only 4.2% of input‑control overlays reached statistical significance. This comparison demonstrates that the strong enrichment seen for TF datasets is not an artifact of background sequencing signal but instead reflects a highly specific association between TFs and G4-prone sequences. Results with input controls are provided in Supplementary Material 03.

## Discussion

Non‑B DNA structures have emerged as important regulatory elements within promoters, where their ability to adopt diverse topologies like triplexes, cruciforms, and G4, can influence transcriptional initiation and chromatin architecture [39, 40]. Many TFs recognize not only specific nucleotide sequences but also structural features of DNA, with p53 representing a prominent example of a protein capable of binding a broad spectrum of non‑canonical DNA topologies [41]. Increasing evidence shows that G4 motifs within promoter regions are particularly relevant modulators of gene expression, affecting TF recruitment, nucleosome positioning, and epigenetic state [42, 43]. Recent work has demonstrated that G4 structures allow nucleosome exclusion and RNA polymerase II pausing at promoters [19], and modulate alternative promoter selection to shape transcript diversity [20]. Interactions between p53 and G4s located in promoters have been demonstrated in several disease contexts, including cancer, where G4 structures can enhance transactivation by both wild‑type and partially functional p53 mutants [44, 45]. Functional studies further illustrate that G4-containing promoter sequences can increase transcriptional output in model systems, highlighting their general regulatory potential [46]. Collectively, these findings underscore the significance of non‑B DNA structures, and G4 in particular, as dynamic modulators of promoter activity with broad implications for cellular homeostasis, metabolic disease, and cancer biology.

Using the G4Hunter algorithm, we found that high-scoring G4 motifs are distributed in a non-random, region-specific manner, with the highest densities on gene-rich chromosomes such as 19 and 16, in contrast to gene-poor chromosomes like 18 and Y [37, 47]. The elevated G4 density on chromosome 19 is a predictable consequence of its exceptionally high GC content and gene density [37], and does not reflect an unexplained biological anomaly. The G4Hunter algorithm inherently accounts for local GC composition in its scoring, and the permutation-based enrichment analyses rely on randomized backgrounds that preserve chromosomal length distributions, jointly ensuring that global CG composition is appropriately accounted for. Features analysis further showed that G4 motifs cluster around regulatory elements, especially before and after exons, genes, and precursor RNA, and within repeat regions. These patterns match recent genome-wide mapping studies demonstrating strong G4 accumulation at promoters, enhancers, UTRs, and splice junctions, and supporting their emerging role as potential therapeutic targets at oncogene promoters [48, 49]. Additional evidence suggests that G4 structures contribute to long-range chromatin interactions and three-dimensional genome organization [50, 51], while feature-specific trends, such as upstream exon enrichment, symmetric boundary enrichment, and downstream localization in precursor RNAs, are consistent with known roles in splicing and polyadenylation [52, 53]. G4s are enriched at topologically associating domain (TAD) boundaries and contribute to chromatin insulation [50], while G4-mediated DNA looping can regulate gene expression at genomic distances [51] – suggesting that many observed TF–G4 associations may reflect indirect recruitment through chromatin architecture rather than direct molecular contact. In contrast, G4 motifs were rarely found in centromeres and heterochromatin, indicating possible selective exclusion from compact genomic environments [54]. It is important to note that G4Hunter-based prediction identifies genomic regions with G4-forming potential: the very high threshold chosen in this study nearly guarantees G4 formation in vitro [10]. This stringent threshold was deliberately chosen to minimize false positives and prioritize specificity over sensitivity, yielding a smaller but highly reliable set of G4 loci for downstream analysis. Importantly, the conclusions of this study are intentionally restricted to high-confidence, strongly G4-prone loci, rather than all possible G4 motifs predicted at lower stringency. The consistent enrichment of BG4 ChIP-seq datasets at sequences predicted by G4Hunter (100% of BG4 datasets enriched), supported the quality of the G4 motif prediction. This is unsurprising, given the very high G4Hunter threshold chosen for this study. By applying this stringent threshold, our analysis focuses on a conservative subset of highly G-rich, G4-prone sequences, which may also represent an accumulation of G4-prone contexts rather than constitutively folded G4 structures. Consequently, the observed TF associations should be interpreted as preferential binding to genomic regions with very strong G4-forming propensity. Of note, these regions are also very prone to i-motif formation when considering the C-rich complementary strand. Given that most, if not all, ChIP-seq experiments are not strand-specific, we cannot exclude that some of the TF colocalize with G4-prone regions via binding to this complementary C-rich strand, folded into an i-motif or not. More broadly, many of the enrichments observed in this study may not reflect direct binding of TFs to G4 structures. G4-forming sequences are frequently found at active gene promoters and regulatory elements, where many transcription factors are expected to bind regardless of G4 presence. Therefore, a proportion of the observed co-localization likely reflects the shared genomic context (active regulatory regions) rather than a specific recognition of G4 or i-motif structures by the TF itself.

Exploring alternative G4Hunter thresholds or extending this analysis to additional species may further refine the relationship between TF binding and G4–TF association. Lowering the G4Hunter threshold (e.g. to 2.0) would be expected to increase sensitivity by including more G4-prone regions, potentially reducing the strongest TF–G4 associations observed here. For this study, we therefore focused on a stringent threshold to prioritize robustness and biological interpretability. This stringent threshold was deliberately chosen to minimize false positives and to prioritize specificity over sensitivity, ensuring that only the sequences with the highest biophysical G4-forming propensity were included in the analysis. Accordingly, our conclusions are intentionally restricted to high-confidence G4 loci and should not be extrapolated to weaker G4-prone sequences detectable at lower score thresholds.

Our findings are broadly consistent with the landmark study by Spiegel et al. [55], which demonstrated that endogenous G4 structures serve as TF binding hubs in human chromatin, analyzing 524 ENCODE-derived datasets in K562 and HepG2 cells. Both studies independently identify NRF1 and SP1 among the most consistently G4-enriched factors: in our dataset, NRF1 showed enrichment in all 41 analyzed datasets (100%, with 35 datasets exceeding 10-fold enrichment – our threshold for strong association), and SP1 was enriched in 35 of 39 datasets (89.7%, with 17 datasets exceeding 10-fold enrichment). This finding is mechanistically supported by a recent study demonstrating that SP1 forms transcriptional condensates at G4-containing promoters in a G4-dependent manner, with G4 structures being required for SP1-mediated transcriptional activation of the Bcl3 oncogene [56]. However, these two studies diverge in several important aspects. First, Spiegel et al. used experimentally mapped endogenous G4s (BG4 ChIP-seq), while we rely on high-stringency computational predictions (G4Hunter ≥ 3.0), capturing a partially distinct G4 set. Second, we analyze a substantially larger compendium – 1,846 unique proteins across 32,817 ChIP-seq datasets – enabling reproducibility assessment across diverse cell types rather than single cell-line conditions. Third, we explicitly characterize proteins consistently depleted from G4 regions, a dimension not systematically addressed in Spiegel et al. Together, our results reinforce and extend the G4 binding hub concept by emphasizing reproducibility and consistency across biological contexts. A dedicated database of G4-interacting proteins (G4IPDB) was not accessible at the time of analysis. We therefore cross-referenced our 1,463 enriched proteins (FE > 1, logP < − 3) against two independently published G4-binding protein reference lists. The first, by Sánchez-Martín et al. [57]., provides a literature-based overview of experimentally validated G4-binding proteins. Of their 31 listed proteins, 23 were represented in the ChIP-Atlas database and could be evaluated; 22 of these 23 (96%) were enriched in our analysis. The second reference, by Zhang, Spiegel et al. [58]., used live-cell chemical proteomics – a photoactivatable G4 ligand combined with UV crosslinking – to identify 256 G4-interacting proteins. This approach captures direct G4–protein contacts in a cellular context, but is inherently dependent on the specific ligand used and the cellular conditions applied, which may not reflect the full repertoire of G4-associated proteins. Of the 256 proteins, 75 were present in ChIP-Atlas and evaluable; 58 of these (77%) were enriched in our dataset. Notably, among the 37 most consistently enriched proteins in our analysis (enriched in > 90% of their datasets, Table 1), four were present in the Sánchez-Martín reference list – YY1, CTCF, TP53, and KDM1A – and one, STAG1, was identified in the Zhang et al. dataset. The convergence between our large-scale computational analysis and these independent experimental approaches supports the validity of our G4-enrichment results.

TFs identified in our analysis have previously documented associations with G4 structures. Among these STAT3 [59], BRD4 [60], TP53 [45, 61] and CTCF [62] represent the most compelling G4‑associated proteins, supported by biochemical evidence, functional assays, and genome-wide studies [55]. In addition to the finding that 100% of BG4 ChIP‑seq datasets showed enrichment at predicted G4 regions (confirming the validity of our approach), STAT3 emerged as particularly noteworthy, showing enrichment in *all* 127 STAT3 ChIP‑seq datasets available in the ChIP‑Atlas resource. This result is consistent with multiple independent reports describing direct binding of STAT3 to G4‑forming DNA and its involvement in G4‑dependent transcriptional regulation [59].

To refine our analysis, we focused on proteins that showed strong (> 10-fold) enrichment at G4-associated sites across a large number of ChIP-seq datasets. A notable example is BRD4, which showed strong and highly consistent enrichment at G4-associated genomic regions. In our analysis, BRD4 was enriched in 690 of 720 datasets (95.8%), with 431 ChIP-seq experiments showing more than a 10-fold enrichment at G4 sites, indicating a strong and recurrent association with G4-rich genomic regions. This observation is consistent with experimental and genome-wide studies demonstrating that BRD4 preferentially localizes to G4-enriched enhancers and super-enhancers, where G4 structures contribute to BRD4-dependent transcriptional activation, enhancer function, and chromatin remodeling. Moreover, biochemical assays have shown that BRD4 can interact with G4 in vitro, supporting a mechanistic link between G4 structures and BRD4 recruitment in chromatin [60].

In addition, TP53, a master tumor suppressor governing DNA damage response, cell cycle arrest and apoptosis [63], was enriched in 96.2% of the examined datasets, with 232 ChIP‑seq experiments showing more than a 10‑fold enrichment at G4 sites. TP53 has been experimentally demonstrated to bind telomeric G4 structures, where it contributes to the maintenance of chromosomal stability [61]. More recently, ChIP experiments in myeloid leukemia cell lines directly confirmed TP53 binding to G4-forming genomic sequences, providing in vivo evidence consistent with our genome-wide enrichment data [45].

CTCF showed a strong association with G4 regions, being enriched in 1,861 of 1,932 datasets (96.3%), including 218 with > 10-fold enrichment. This frequent co-localization is consistent with reports that G4 motifs often occur near CTCF binding sites, where they may enhance binding affinity and support chromatin looping events that regulate gene expression [62]. Notably, G4 structures have been shown to directly promote CTCF recruitment – both through R-loop-associated G4 formation [62], and through direct G4 binding by CTCF itself [64], with the latter regulating CTCF occupancy, chromatin looping and gene expression genome-wide. CTCF functions both as a direct transcriptional regulator at promoters and as a key organizer of three-dimensional chromatin structure [65, 66], and its frequent co-localization with G4 motifs may therefore reflect involvement in both of these roles.

Collectively, the top-scoring enriched proteins identified in this study – STAT3, BRD4, TP53, and CTCF – are not generic chromatin-associated proteins but factors with well-defined biological roles that are mechanistically consistent with preferential localization to G4-rich regulatory regions.

Interestingly, we also identified several TFs not traditionally recognized G4-associated factors such as FLI1, REST, SUPT5H, HSF1, ESR1 and NR3C1, which repeatedly co‑localized with G4‑rich regions across many ChIP‑seq datasets. FLI1 was enriched in 82/83 datasets (25 with > 10‑fold enrichment), and its oncogenic fusion, EWS–FLI1, is regulated by G‑rich RNA elements capable of forming G4, suggesting context‑dependent involvement [67]; notably, FLI1 is not listed among canonical / experimentally validated G4-binding proteins [26, 57]. REST was enriched in 118 of 120 datasets, consistent with reports that G4 structures have been suggested to recruit the REST–LSD1 repressive complex, although direct G4 binding by REST remains unconfirmed [57]. HSF1 (101/105 datasets; highest median FE) and SUPT5H (84/87 datasets, with variable high‑confidence signals) are likewise not listed among canonical G4‑binding proteins in key reviews, and no direct molecular interaction with G4 DNA has yet been demonstrated for either [26, 57]. ESR1 also showed a strong and consistent co-localization with G4-rich regions, with enrichment observed in 94.8% of datasets and 511 experiments exceeding the 10-fold threshold. Consistent with genome-wide analyses identifying ESR1 as a non-canonical factor enriched at G4-positive regulatory loci rather than a direct G4 binder, this pattern supports an indirect or context-dependent association of ESR1 with G4-associated chromatin [55]. The NR3C1 protein (glucocorticoid receptor) showed strong enrichment (374/401 datasets). NR3C1 is a ligand-activated TF with a well-established role in glucocorticoid-dependent gene regulation [68]. Although NR3C1 has not been previously implicated in G4 biology [26, 57], our results suggest a potential association with G4-rich regulatory regions, pointing to a previously underappreciated link between glucocorticoid signaling and G4-associated regulation. This enrichment likely reflects the preferential binding of NR3C1 to active regulatory regions with high G4 density, rather than a specific G4-dependent mechanism within the glucocorticoid signaling pathway. Whether this co-localization has functional consequences for glucocorticoid-responsive gene regulation remains to be determined experimentally. It should be noted that ChIP-seq datasets may contain non-targeted transcription factor binding as a systemic component [69], and therefore co-localization observed for non-canonical candidates such as REST, HSF1, NR3C1, and FLI1 should be interpreted as potential association with G4-rich regions rather than direct G4 interaction.

Our analysis reveals that G4-prone motifs may serve as regulatory hotspots for a far broader set of TFs than previously recognized, with many non‑canonical candidates, including REST, NR3C1, FLI1, HSF1 and SUPT5H, showing frequent and consistent co‑localization with G4‑rich loci across ChIP‑seq datasets, as summarized in Table 1 and Supplementary Material 02. G4-enriched TFs identified in this study are predominantly associated with GC-rich regulatory elements, transcriptional activation, and chromatin remodeling, consistent with the established role of G4 structures as positive regulators of transcription [70, 71].

At the same time, a distinct subgroup of TFs, such as ZNF24, FOXK2, HMGA1, CTBP1, NKX2‑1 and GATA4 consistently avoided G4 regions, likely reflecting inherent sequence preferences (e.g., A/T‑rich binding motifs) or chromatin contexts incompatible with G4 formation. For example, HMGA1 has preference for A/T‑rich DNA via AT‑hook motifs [72] and FOXK2, a member of the forkhead family, shows preference for A/T‑rich sequences [73]. More broadly, the canonical forkhead binding motif RYAAAYA is shared across most FOX family members [74], suggesting that avoidance of G-rich G4-forming sequences may be a general property of the forkhead family rather than specific to FOXK2. A systematic analysis of the relationship between A/T content of TF binding motifs and G4 enrichment or depletion would be an informative direction for future work. Regions with strong G4-forming potential may therefore be structurally inaccessible to TFs that require linear B-form DNA for binding, explaining their consistent depletion from G4-rich loci.

Another interesting group of proteins is the NF-κB family, which functions as either homodimers or heterodimers composed of different subunits, several of which have been associated with G4 structures. Key subunits include p50 (NFKB1), p52 (NFKB2), and p65 (RelA). Among these, NFKB1 was enriched in all 25 of its datasets, 21 of which showed more than 10-fold enrichment. NFKB2 was enriched in all 35 of its datasets, while RelA was enriched in 249 of its 268 datasets (93%; Supplementary material 03). The NF-κB pathway itself is a well-characterized oncogenic signaling pathway involved in melanoma tumorigenesis and progression [75]. Proteomic studies have shown that activation of the JAK/STAT3 pathway facilitates the translocation of RelA to mitochondria, where it binds mitochondrial DNA G4s, promotes their folding, and consequently increases mtDNA instability, inhibits mtDNA transcription, and leads to mitochondrial dysfunction [76]. Additionally, the cytosolic RNA helicase DDX41 binds DNA and activates STING, ultimately leading to activation of NF-κB and interferon regulatory factor 3 (IRF3). G4s therefore may act as a molecular signal through interactions with multiple DNA- and RNA-binding proteins [77].

**Fig. 6:**
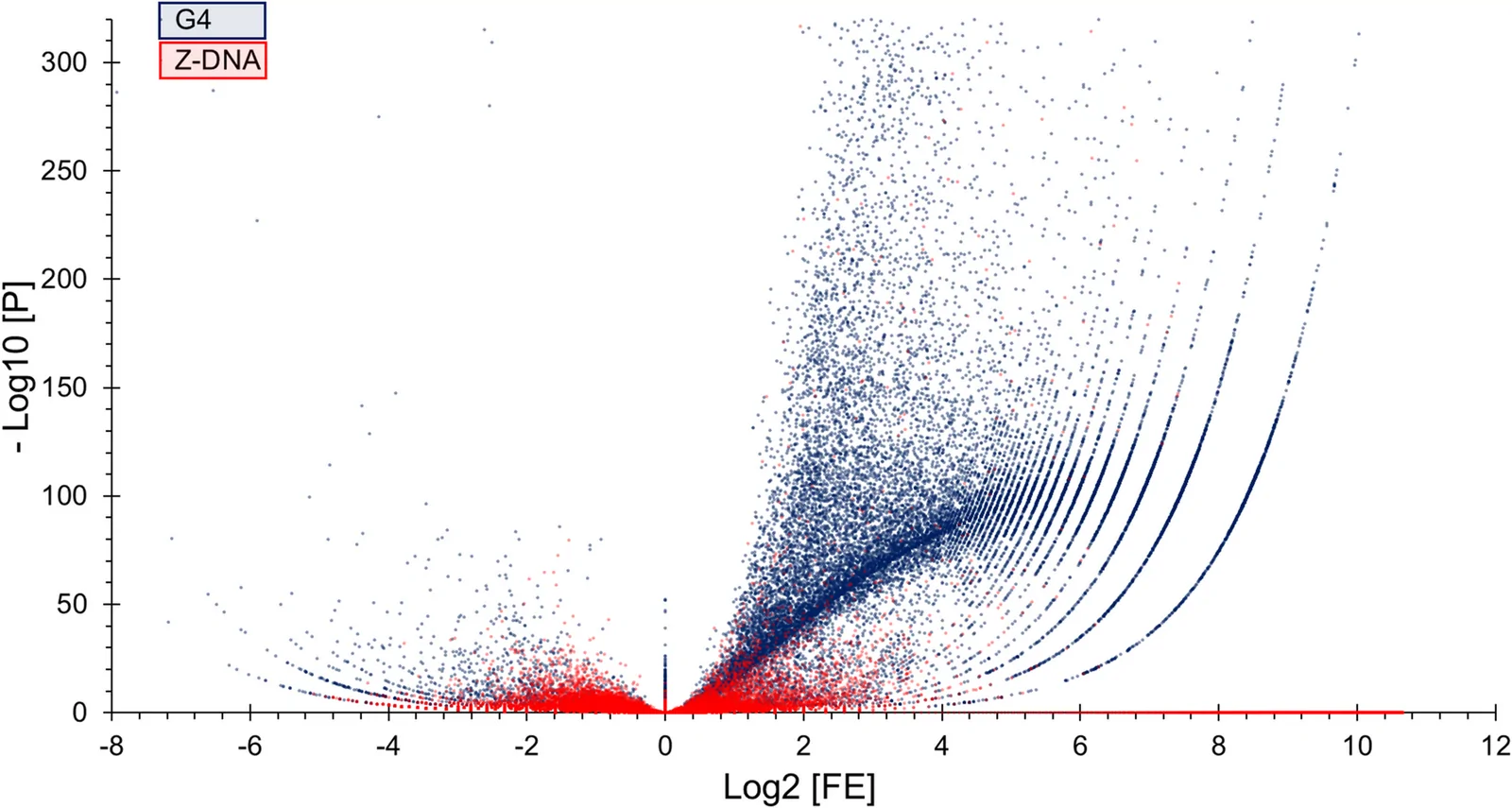
Volcano plot of proteins association in G4-forming sequences (blue) and Z-DNA (red) regions (dots above 0 – abundance, below 0 – depletion). The strongest abundance is shown in right upper part of the plot

A comparative analysis of proteins enriched at G4 and Z-DNA associated regions [78] revealed notable differences in binding patterns (Fig. 6). Across all analyzed proteins, enrichment at G4-forming sequences was far more widespread than at Z-DNA forming sequences (ZFS): 91% of proteins were enriched at G4-forming sequences compared with only 32% at ZFS, and strong enrichment thresholds (FE > 2, 5, 10) were consistently reached by a much larger fraction of G4-associated proteins than ZFS-associated ones. Although several proteins with numerous ChIP-seq entries, such as MAX, ARNT, TP53, MCM2, AHR, MNT, and FOXP1, showed enrichment at ZFS in 100% of their analyzed ChIP-seq datasets, their enrichment at G4-forming sequences was more variable, whereas proteins strongly enriched at G4-forming sequences (including STAT3, ARID1A, YAP1, SMARCC1, CDK9, EGR1, KDM5B) often showed weak or inconsistent association with Z-DNA. Only a small subset of factors like STAT3, CREB1, ARNTL, NRF1, and EPAS1, showed enrichment in 100% of their ChIP-seq datasets in both G4 and ZFS contexts, suggesting a broader affinity for non-B DNA structures. These comparisons demonstrate that although ZFS recruit a narrow and specific cohort of TFs, G4-forming sequences attract a much larger and more diverse set of transcriptional regulators, while selectively excluding others. Overall, the prevalence and breadth of TF enrichment at G4-forming sequences indicate that G4-forming sequences represent a more general and widely used regulatory feature of human promoters than Z-DNA forming regions, supporting their central role in shaping TF occupancy and genome regulation.

Our findings suggest that G4 motifs might act as structural hubs for a broader array of TFs than previously assumed. While established regulators like STAT3 and MYC show clear associations, we also observed consistent co-localization of several other proteins with G4 regions, even though direct experimental confirmation is still lacking for many of them [69]. Accordingly, the associations reported here reflect reproducible genomic co‑localization patterns rather than direct motif‑driven TF–DNA recognition. This pattern points to the possibility that G4-driven gene regulation plays a more extensive role than currently documented, highlighting the need for further experimental follow-up. Altogether, these observations suggest that G4 structures may act as recruitment platforms for certain proteins, and as structural barriers for others. The chosen threshold for G4 identification (> 3) is very high, retaining only highly G4-prone sequences with a strong propensity to form stable G4 structures, acting as strong structural barriers. In contrast, some TFs may naturally be excluded from these regions. As a result, G4-depleted TFs could represent a functionally distinct group that operates through alternative regulatory mechanisms.

These findings extend the current landscape of G4 biology and highlight specific proteins that justify closer functional investigation. It should be noted that our analysis focuses on enrichment consistency across datasets (presence/absence) rather than quantitative peak intensities. Correlating G4Hunter scores with ChIP-seq peak heights or TF motif recognition scores would provide additional mechanistic insight. However, such analyses require harmonized, quantitative ChIP-seq data that are not uniformly available through ChIP-Atlas. We therefore frame this as an important direction for future targeted studies. Restricting analyses to top-ranked ChIP-seq peaks (e.g. top 1,000–5,000 by signal) could further emphasize higher-affinity binding events and represents a complementary approach for future investigations. Exploring the roles of these G4-associated proteins in G4-associated regulation and transcriptional modulation may uncover unexpected regulatory pathways and provide novel insight into genome organization, stress response, and disease-related processes. Furthermore, expression breadth – including the distinction between housekeeping and tissue-specific genes as defined by the Human Protein Atlas [79] – represents an important dimension for future interpretation of G4-associated TF regulation. Throughout this analysis, we treat enrichment as evidence of genomic co-localization rather than direct molecular interaction; establishing causality requires targeted experimental validation for individual proteins. Experimental validation of individual TF–G4 interactions is beyond the scope of this computational meta-analysis. Our study is intended as a systematic discovery and prioritization resource, identifying candidates for targeted follow-up. Notably, several top-ranked proteins identified here – including STAT3, BRD4, TP53, and CTCF – already have strong independent experimental support for direct G4 interaction, providing confidence in the overall approach.

Importantly, our conclusions pertain to global and reproducible TF–G4 associations rather than cell-type-specific regulatory events. The ChIP-Atlas resource aggregates data across a wide range of cell types and conditions, and our analytical strategy deliberately leverages this heterogeneity: by requiring consistent enrichment across many independent datasets, cell types, and experimental contexts, we specifically aim to identify robust, cell-type-independent patterns rather than associations driven by a single biological condition. To illustrate the cross-tissue robustness of these associations, we performed a cell-type-stratified analysis for some representative TFs included in our tissue distribution analysis. As shown in in Supplementary material 05, TFs with broad regulatory roles – including BRD4, TP53, CTCF, REST, NR3C1, HSF1, STAT3, and SP1 – show significant G4 enrichment across 8–19 distinct cell classes, demonstrating that their association with G4 structures is not confined to a single cellular context. In contrast, tissue-restricted factors displayed the expected cell-type specificity: ESR1 enrichment was concentrated predominantly in breast-derived datasets (88%), consistent with its role as the estrogen receptor, while FLI1 enrichment was observed mainly in bone marrow and blood (87%), reflecting its function in hematopoiesis. These patterns confirm that the observed TF–G4 co-localization signals reflect intrinsic TF–G4 affinity modulated by tissue-specific expression, rather than a systematic bias toward any single cell type. Dissecting cell-type-specific G4–TF interactions in greater detail represents an important future direction, which could be addressed using matched G4 mapping and ChIP-seq datasets in defined cellular systems.

To further characterize the functional identity of proteins showing consistent G4 co-localization versus those depleted at G4 sites, we performed Gene Ontology (GO) overrepresentation analysis across all three major annotation categories, namely biological process, molecular function, and cellular component, using the PANTHER classification system. Gene Ontology overrepresentation analysis of the high-enrichment protein group identified several GO Biological Process terms with fold enrichment values exceeding 75. The most prominently enriched terms included processes associated with renal development, such as regulation of metanephric glomerular mesangial cell proliferation (GO:0072301) and negative regulation of mesenchymal to epithelial transition involved in metanephros morphogenesis (GO:0003340), alongside immune cell differentiation (follicular dendritic cell differentiation; GO:0002268), TGF-β signaling (positive regulation of transforming growth factor beta3 production; GO:0032916), and chromatin-related cellular responses to trichostatin A (GO:0035983, GO:0035984). Post-translational modification and antiviral processes were also represented. The extreme fold enrichment values observed for these terms reflect complete overlap between the input list and the reference annotation, as each term annotated only 2–3 genes in the reference genome, all of which were present in the high-enrichment dataset.

Overrepresentation analysis of the depleted protein group within the GO Biological Process category revealed a markedly different functional profile compared to the high-enrichment group. The most significantly enriched terms were predominantly associated with transcriptional repression, including negative regulation of DNA-templated transcription (GO:0045892), negative regulation of RNA biosynthetic process (GO:1902679), negative regulation of RNA metabolic process (GO:0051253), and negative regulation of transcription by RNA polymerase II (GO:0000122), with highest fold enrichment values ranging from 13.24 to 14.75 and FDR-adjusted *p*-values below 0.001. The consistent overrepresentation of transcriptional repression terms across multiple related GO categories suggests that certain proteins showing low overlap with ChIP-Atlas data at G4 sites are functionally associated with gene silencing and repression of RNA synthesis.

GO molecular function analysis of the high-enrichment group revealed strong overrepresentation of chromatin-associated activities, including histone acetylation reader activities (GO:0140109, GO:0140012, GO:0140011), histone H3K27 acetyltransferase activity (GO:0044017), acetylation-dependent protein binding (GO:0140033), and cohesin loader activity (GO:0061775), all with fold enrichment values over 75 and FDR below 0.05. This pattern is consistent with the predominant role of G4-associated proteins in chromatin remodeling and epigenetic regulation [80, 81].

In contrast, the depleted group was overrepresented for terms related to sequence-specific DNA binding and transcriptional activation, including RNA polymerase II cis-regulatory region sequence-specific DNA binding (GO:0000978), DNA-binding transcription activator activity (GO:0001216), and transcription factor binding (GO:0008134), with fold enrichment values ranging from 13.4 to 26.06. This suggests that proteins with low G4-ChIP overlap include classical sequence-specific transcription factors that bind canonical promoter elements independently of G4 structures.

GO Cellular Component analysis of the highly enriched proteins revealed strong overrepresentation of specific transcriptional and chromatin regulatory complexes, all with fold enrichment values > 75. Enriched terms included the Scc2-Scc4 cohesin loading complex (GO:0090694) and SMC loading complex (GO:0032116), consistent with the cohesin loader activity identified in the Molecular Function analysis, as well as transcription regulatory complexes including the ISGF3 complex (GO:0070721), NF-κB p50/p65 complex (GO:0035525), Bcl3/NF-κB2 complex (GO:0033257), and C/EBP complex (GO:1990647). This pattern suggests that G4-associated proteins are often components of transcriptional protein complexes involved in immune signaling and chromatin organization.

In contrast, the depleted group showed overrepresentation of broader transcriptional regulatory assemblies, including the transcription regulator complex (GO:0005667; fold enrichment > 25) and RNA polymerase II transcription regulator complex (GO:0090575; fold enrichment around 25), consistent with the Molecular Function findings and further supporting that proteins with low G4-ChIP overlap are predominantly components of the general RNA polymerase II transcriptional machinery rather than G4-associated specialized chromatin complexes.

## Methods

### Data acquisition

The genomic data used in this study were sourced from the National Center for Biotechnology Information (NCBI). We downloaded the current telomere-to-telomere assembly of human genome (T2T-CHM13v2.0) to G4-forming sequences prediction and subsequently converted results by Liftover (NCBI) [82] to GRCh38 assembly coordinates to ensure compatibility to the more complete annotations of human genome provided by broadly used GRCh38 assembly compatible with ChIP-Atlas annotations. Data on TF binding enrichment were obtained from ChIP-Atlas, a publicly available platform that compiles and processes large-scale ChIP-seq experiments [35].

### Prediction of G4s

G4-prone sequences were identified using G4Hunter, an established algorithm that estimates the probability of G4 structure formation based on local guanine content [10]. Higher G4Hunter scores reflect a stronger propensity of a given sequence to form a G4, and this G4 is expected to be more stable. The analysis was performed genome-wide across all human chromosomes using the G4Hunter web application (https://bioinformatics.ibp.cz) [36]. Predictions were generated using a 25-nucleotide sliding window and a stringent score threshold of ≥ 3.0. This high threshold was chosen to limit the high genomic abundance of lower-scoring G4 predictions, which would otherwise lead to widespread and largely unspecific overlap with the extensive ChIP-seq datasets used in this study. By focusing on a reduced set of highly G-rich sequences, this approach minimizes background overlap while preserving statistically robust and biologically meaningful associations with transcription factor binding. Predicted G4 sites were exported ungrouped in BED format for later computational analysis. Total counts obtained by summing chromosome-specific outputs, and summary statistics, are available in Supplementary Material 01.

### Analysis of G‑quadruplex–forming sequences around annotated features

The feature table containing functional annotations for the GRCh38 reference genome assembly was downloaded from the NCBI database. Annotated genomic features were grouped according to their classification, and metadata describing their genomic coordinates and lengths was retained for downstream analysis. The occurrence of predicted G4-forming sequences was quantified within each annotated feature, as well as in the 100 bp regions upstream and downstream of those features.

### ChIP-atlas: enrichment analysis

To investigate shared regulatory or epigenetic features associated with specific TF sets, we performed enrichment analysis using ChIP-Atlas, a comprehensive database that integrates large-scale ChIP-seq datasets (https://chip-atlas.org/). The “TFs and others” experiment category, comprising 32,817 datasets, was selected for the analysis. This category encompasses not only canonical sequence-specific TFs but also chromatin-associated regulators and cofactors. Throughout this study, the term ‘protein’ is used where appropriate to reflect this broader composition, and results should be interpreted accordingly. Curated TF-specific classification is available through resources such as GTRD [83], which integrates TFClass and AnimalTFDB annotations for each factor; however, such re-annotation was not applied in the present analysis. All cell type classes available in ChIP-Atlas were included to maximize dataset coverage. A significance threshold of 100 was applied to identify TFs exhibiting statistically enriched binding within the G4 regions. All ChIP-seq peaks provided by ChIP-Atlas, uniformly processed from MACS2 outputs, were used without additional peak-rank filtering. This approach was chosen to ensure consistency across the > 32,000 datasets, which originate from diverse cell types, antibodies, and experimental designs; applying top-ranked peak thresholds uniformly across such heterogeneous data would risk introducing systematic bias. Our analysis does not restrict ChIP-seq peaks to 5′ regions but evaluates enrichment relative to predicted G4-forming regions genome-wide, capturing both promoter-proximal and distal regulatory contexts. Promoter-restricted analyses would represent a complementary but separate line of investigation. G4 regions of interest were obtained from G4Hunter output and supplied to ChIP-Atlas as BEDGRAPH files with 100 permutation iterations, in which each genomic interval is randomly repositioned across the genome to generate a randomized background model. This randomization preserves the chromosomal length distribution of the input regions, ensuring that the background model accounts for global GC and sequence composition at the chromosome level. To enhance background quality and statistical robustness, 100 permutation iterations were used to generate a randomized background model. Since ChIP-Atlas provides exclusively peak regions, non-peak genomic regions required for a standard contingency-table analysis (*e.g*. Fisher’s exact test) are not available within this framework. Enrichment significance is therefore assessed at the dataset level through permutation-based comparison rather than simple peak overlap. The number of peaks and fold enrichment values were taken directly from the ChIP-Atlas enrichment analysis output. Enrichment was defined as fold enrichment > 1, and statistical significance as log *P* < − 3. A stringent significance threshold of *p* < 0.001 was applied across all datasets to minimize false positives. ChIP-Atlas additionally reports a significance value that is already corrected for the number of datasets tested (a Benjamini-Hochberg FDR q-value). Every dataset we identified as significant, whether enriched or depleted, also passed this corrected threshold (*q* < 0.05), confirming that the reported associations are not an artifact of multiple testing. A complete list of proteins enriched and depleted in G4 regions, along with summary statistics, is available in Supplementary material 02. To address potential confounding by promoter enrichment – since both TF binding and G4 motifs are enriched at promoters – our strategy relies on consistency of enrichment across many independent datasets from diverse cell types, conditions, and experimental designs. TFs showing enrichment across this breadth of contexts are unlikely to represent a trivial artifact of promoter accessibility alone. This deliberate inclusion of all available cell type classes in ChIP-Atlas is an analytical strategy: by requiring consistent enrichment across many independent datasets, cell types, and experimental conditions, we specifically aim to identify robust, cell-type-independent TF–G4 associations rather than patterns driven by a single biological context.

### Gene ontology analysis

Proteins identified as enriched through ChIP-Atlas analysis were subjected to GO biological process overrepresentation analysis using the PANTHER classification system (https://pantherdb.org/). To provide a complete functional characterization, the analysis was performed across all three major GO categories - biological process, molecular function, and cellular component. Two protein sets were defined based on ChIP-Atlas enrichment criteria:

a high-enrichment group comprising 279 proteins present in more than 10 ChIP-Atlas datasets with an enrichment score exceeding 90%, and a low-enrichment control group of 10 proteins present in more than 10 datasets but with an enrichment score of 20% or less. Each set was independently mapped against the complete *Homo sapiens* reference proteome (20,580 genes), which served as the statistical background for all comparisons. Overrepresentation of GO biological process terms was assessed using Fisher’s exact test, whereby the frequency of each GO term within the input list was compared to its expected frequency in the reference genome. Resulting *p*-values were corrected for multiple testing using the Benjamini-Hochberg false discovery rate (FDR) procedure, and only GO terms with an FDR-adjusted *p*-value below 0.05 were considered statistically significant and retained for further interpretation. Fold enrichment, defined as the ratio of observed to expected term frequency, was used as a measure of the magnitude of overrepresentation, reflecting how much more frequently a given GO term appeared in the input list relative to random expectation based on the reference proteome (Supplementary Material 04).

### Statistical analysis

The statistical evaluation of G4-forming sequences distribution across human chromosomes, was performed by the interquartile range (IQR) method to mark potential statistical outliers. We calculated the first (Q1) and third (Q3) quartiles, denoted as Q1 = 3.21, Q3 = 4.31, which gave us an IQR value of 3.58. Any chromosomes with G4 frequencies below and over Q1 and Q3 were interpreted as having significantly reduced G4 content compared to the genome-wide trend. To explore whether G4 site distributions approximate normality across chromosomes, we applied the Tukey test. For ChIP‑Atlas enrichment analyses, only highly statistically significant results (*p* < 0.001) were considered for downstream interpretation and presentation.

## Conclusion

This study provides a genome‑wide characterization of G4-forming sequences and their associated transcriptional regulators, revealing that G4-forming sequences are unevenly distributed across the human genome and preferentially accumulate in highly active regulatory regions such as promoters, introns, repetitive sequences, UTRs etc. By integrating predicted G4 loci with more than 32,000 ChIP‑seq datasets, we identified strong and selective co‑localization between G4-forming sequences and a distinct subset of TFs. Canonical G4-binding proteins, including STAT3, TP53, MYC, NRF1, CTCF, and BRD4, showed consistent enrichment, validating their established roles in G4-associated regulation. Importantly, our approach also revealed several promising TFs not previously linked to G4 biology, such as REST, NR3C1, FLI1, HSF1 and SUPT5H, each displaying recurrent overlap with G4-forming sequences across independent datasets. These findings expand the repertoire of potential G4-associated regulators and suggest broader regulatory mechanisms involving both direct and indirect engagement with G4s. Our results support a model in which G4s serve either as selective regulatory scaffolds or “DNA roadblocks” that shape transcription-factor occupancy and contributes to gene regulatory specificity, influencing chromatin accessibility and transcriptional output. The high reproducibility of enrichment across diverse datasets underscores the robustness of these associations and highlights the widespread involvement of G4 structures in transcriptional regulation. This integrated analysis provides a systematic resource for future functional studies aimed at dissecting the mechanistic roles of G4-associated TFs in genome regulation. Motif-resolved and structure-resolved analyses of individual candidates identified here represent a necessary next step toward mechanistic interpretation of TF–G4 relationships. Comparative analyses with Z-DNA–associated proteins further emphasize that G4 structures are associated with a partially overlapping but functionally distinct set of proteins, highlighting DNA topology as a determinant of TF targeting. Future studies employing harmonized, quantitative ChIP-seq datasets will be needed to assess whether G4 stability scores correlate with ChIP-seq peak heights or TF motif recognition scores, which may provide further mechanistic insight into the selectivity of G4-associated TF recruitment. At the same time, several factors such as ZNF24 and FOXK2 were consistently depleted from G4-forming sequences, suggesting that G4s act not only as sites of preferential TF co-localization but also as exclusion zones for specific protein classes. Systematic annotation of the identified proteins using resources such as GTRD [83], AnimalTFDB [84], TFClass, or JASPAR [85, 86] would substantially refine TF-specific classification and facilitate prioritization of candidates for future functional studies.

## Supplementary Information

Supplementary Material 1. Summary statistics of identified G4 regions.

Supplementary Material 2. A complete list of proteins enriched and depleted in G4 regions, along with summary statistics.

Supplementary Material 3. A complete list of proteins identified in Z-DNA regions and input control, along with summary statistics.

Supplementary Material 4. Full gene ontology overrepresentation analysis across all annotation categories for G4-enriched and G4-depleted protein sets.

Supplementary Material 5. Cell-type distribution of significant G4 enrichment across representative transcription factors.

## Data Availability

All datasets analysed during the current study are publicly available. Data sources, accession numbers and analysis parameters are provided in the Methods section and Supplementary Materials.

## Abbreviations

ChIP: seq–chromatin immunoprecipitation followed by sequencing
FE: fold enrichment
G4: G–quadruplex
BG4: G–quadruplex–specific antibody
IQR: interquartile range
TF: transcription factor
UTR: untranslated region
ZFS: Z–DNA–forming sequence
