# Supplementary material for: G‑quadruplexes as regulatory platforms for human transcription factors: insights from ChIP-seq data
Source: BMC Genomics. 2026 Jul 29;27:920. doi: 10.1186/s12864-026-13217-5 (PMC13737716; doi:10.1186/s12864-026-13217-5)
Supplement: Supplementary file 1 [file 12864_2026_13217_MOESM5_ESM.pdf]

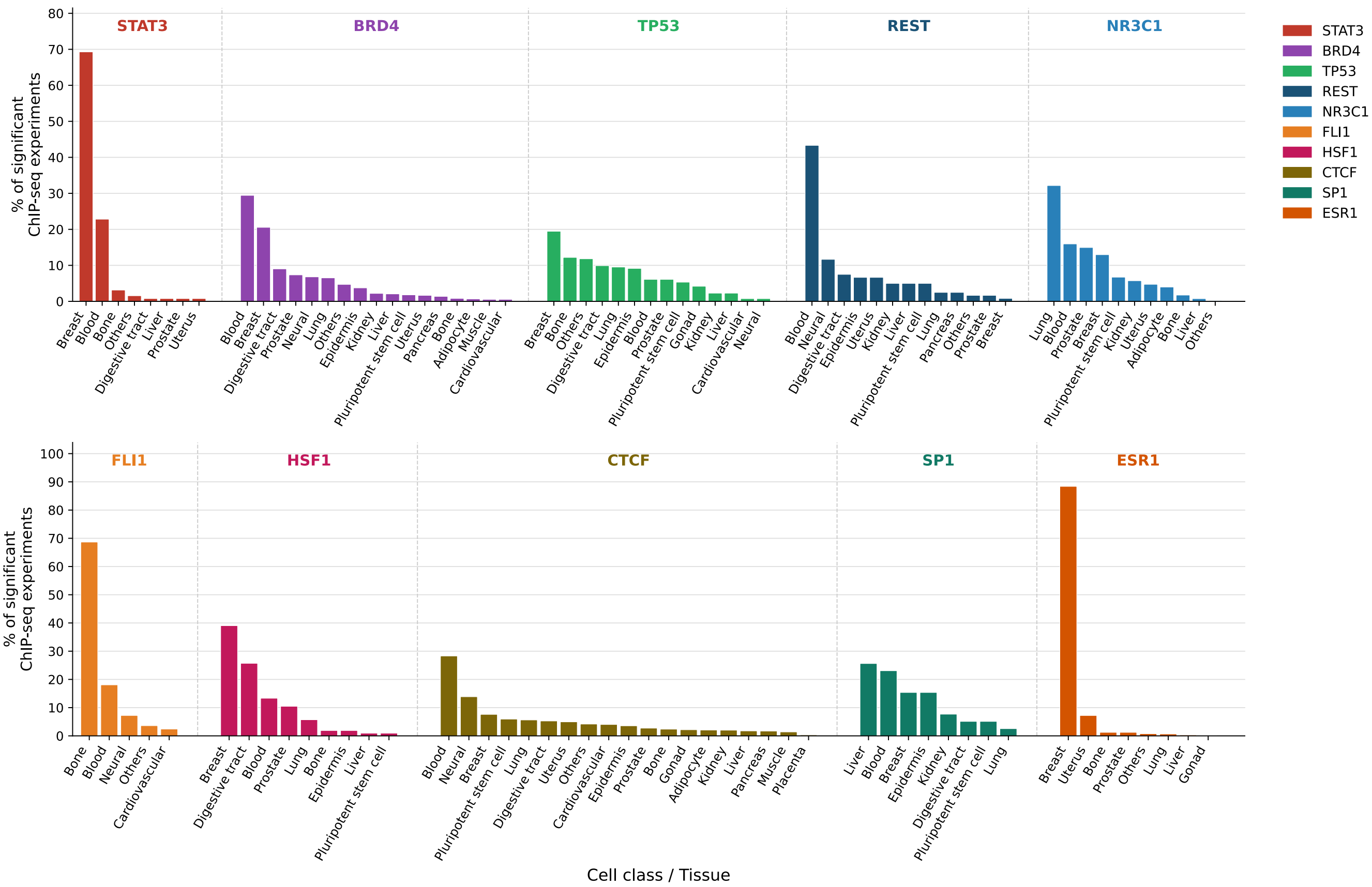

Supplementary material 05: Cell-type distribution of significant G4 enrichment across representative transcription factors. *Bar charts showing the percentage of statistically significant ChIP-seq datasets (p < 0.001) per cell class for 10 selected proteins: STAT3, BRD4, TP53, REST, NR3C1, FLI1, HSF1, CTCF, SP1, and ESR1. For each protein, only cell classes with at least one significant dataset are shown. Percentages are calculated relative to the total number of significant datasets per protein. Data are derived from ChIP-Atlas (SM_03). TFs with broad regulatory roles (BRD4, TP53, CTCF, REST) show enrichment across 8–19 distinct cell classes, whereas tissue-restricted factors show the expected specificity: ESR1 is predominantly enriched in breast-derived datasets (88%), and FLI1 in bone marrow and blood (87%).*
